# Detection of Early Ischemic Changes in Noncontrast CT Head Improved with “Stroke Windows”

**DOI:** 10.1155/2014/654980

**Published:** 2014-03-09

**Authors:** Shraddha Mainali, Mervat Wahba, Lucas Elijovich

**Affiliations:** ^1^Department of Neurology, University of Tennessee, Memphis, TN 38163, USA; ^2^Department of Neurosurgery, Semmes-Murphey Neurologic and Spine Institute, University of Tennessee, Memphis, TN 38120, USA; ^3^Semmes-Murphey Clinic, 6325 Humphreys Boulevard, Memphis, TN 38120-2300, USA

## Abstract

*Introduction*. Noncontrast head CT (NCCT) is the standard radiologic test for patients presenting with acute stroke. Early ischemic changes (EIC) are often overlooked on initial NCCT. We determine the sensitivity and specificity of improved EIC detection by a standardized method of image evaluation (Stroke Windows). *Methods*. We performed a retrospective chart review to identify patients with acute ischemic stroke who had NCCT at presentation. EIC was defined by the presence of hyperdense MCA/basilar artery sign; sulcal effacement; basal ganglia/subcortical hypodensity; and loss of cortical gray-white differentiation. NCCT was reviewed with standard window settings and with specialized Stroke Windows. *Results*. Fifty patients (42% females, 58% males) with a mean NIHSS of 13.4 were identified. EIC was detected in 9 patients with standard windows, while EIC was detected using Stroke Windows in 35 patients (18% versus 70%; *P* < 0.0001). Hyperdense MCA sign was the most commonly reported EIC; it was better detected with Stroke Windows (14% and 36%; *P* < 0.0198). Detection of the remaining EIC also improved with Stroke Windows (6% and 46%; *P* < 0.0001). *Conclusions*. Detection of EIC has important implications in diagnosis and treatment of acute ischemic stroke. Utilization of Stroke Windows significantly improved detection of EIC.

## 1. Introduction 

Noncontrast head CT (NCCT) is the first-line diagnostic test for emergency evaluation of acute stroke due to its speed of imaging, widespread availability, and low cost. The window width and center level settings—measured in Hounsfield units: HUs—used for computed tomographic (CT) scan review are known to influence both lesion conspicuity and diagnostic accuracy [1]. Numerous studies suggest that detection of early ischemic change (EIC) on NCCT can predict both functional outcome and the risk of intracranial hemorrhage (ICH) [2–4].

Specific features relevant to stroke assessment include hyperdense middle cerebral artery (MCA)/basilar signs, focal parenchymal hypoattenuation (notably of the insular ribbon or lenticular nuclei for MCA infarcts), and cerebral swelling manifested by sulcal or ventricular effacement or loss of cortical grey-white differentiation [5–7]. Decreases in CT attenuation accompanying early stroke are small; therefore, their conspicuity may be increased by using narrow window settings centered at approximately the mean attenuation in HUs of gray and white matter. We believe that the increase in lesion conspicuity achieved with this method can improve the accuracy of nonenhanced CT stroke detection [1]. In most academic stroke centers, on-call residents, radiologists, and ED physicians are the first providers to interpret NCCT in the setting of suspected acute ischemic stroke. We hypothesize that detection of EIC can be improved by a standardized method of image evaluation that can be implemented by the treating physicians (including trainees).

## 2. Materials and Methods

### 2.1. Study Description

A retrospective chart review of the University of Tennessee Health Science Center prospective database of acute ischemic stroke from January 2012 to July 2012 was performed. A total of 50 patients with NIH Stroke Scores (NIHSS) greater than 4 were identified who underwent NCCT within 4.5 hours of symptom onset. NCCT with significant motion artifacts and patients with NIHSS less than or equal to 4 were excluded from the study. Nonenhanced CT scanning was performed with a CT HiSpeed Advantage 16 slice scanner (GE Medical Systems, Milwaukee, WI) in the hospital emergency department using the nonhelical scanning technique: 120 KV, 300 mA, 1-second scanning time, and 5 mm section thickness.

At the time of image interpretation, the neurology resident was blinded to all patient information except for the reason of obtaining NCCT (which, in most cases, was the presenting complaint) in order to make the image comparable to the information available to the interpreting radiologists. Images were reviewed at a PACS workstation. Patients' name, sex, and date of birth were visible on the PACS screen (by default) to both the senior resident and the interpreting radiologist.

NCCTs were evaluated for evidence of EIC, which was defined by the presence of one or more of the following findings [5–7]: (1) hyperdense MCA/basilar artery sign; (2) sulcal effacement; (3) basal ganglia/subcortical hypodensity; and (4) loss of cortical gray-white differentiation. Images were first reviewed at the standard settings of 35 HU center and 100 HU width level. Following this, window settings were adjusted to width of 30 HU and center level of 35 HU, which was designated as Stroke Windows. Final interpretation of images was made by neurology resident based on the findings noted on Stroke Windows.

For each patient, the resident's final interpretation using Stroke Windows was compared with the final staff radiologist's report. Accuracy of diagnosis and identification of stroke mimics was then confirmed by review of the final discharge diagnosis. Correlative modalities used to determine the accuracy of EICs included follow-up NCCT and MRI. Statistical analysis was performed using Fisher's exact score.

### 2.2. Standard Protocol Approvals, Registrations, and Patient Consents

This study was approved by the IRB as the part of the University of Tennessee Health Sciences Center's Acute Ischemic Stroke Registry.

## 3. Results

A total of 50 patients were identified; 21 patients were females (42%) with a mean age of 75.4 years; the NIHSS ranged from 5 to 27 with a mean of 13.4. Mean time to CT from symptom onset was 97.7 minutes. Thirty-nine patients had large vessel anterior circulation stroke; 3 patients had posterior circulation stroke; 7 patients had lacunar stroke; 1 patient had no stroke on follow-up imaging. The detection of EICs with Stroke Windows was significantly higher: 70% (35 out of 50 patients) compared to 18% (9 out of 50 patients) in the standard window group (*P* < 0.0001). Rate of detection of individual components of EIC is depicted in Table 1.

**Figure 1:**
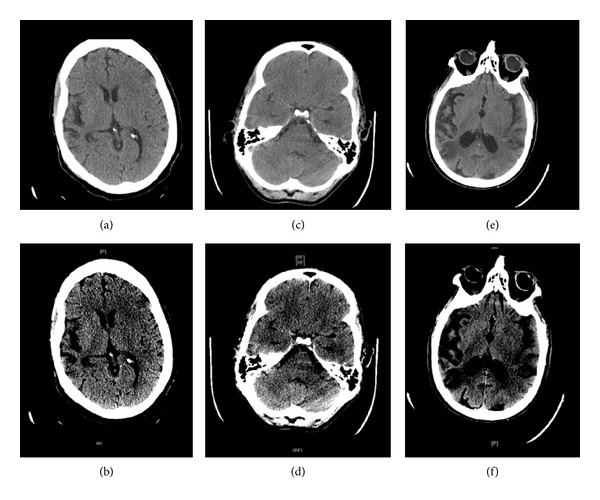
Noncontrast head CTs (NCCTs). (a) NCCT of head viewed on standard windows (35/100). (b) NCCT of head viewed on Stroke Windows (35/30) demonstrating left sulcal effacement of the insular ribbon and hypodensity of left basal ganglia. (c) NCCT of head viewed on standard windows (35/100). (d) NCCT of head viewed on Stroke Windows (35/30) demonstrating right MCA sign. (e) NCCT of head viewed on standard windows (35/100). (f) NCCT of head viewed on Stroke Windows (35/30) demonstrating hypodensity of left basal ganglia.

Hyperdense MCA sign was seen in 18 patients using Stroke Windows, whereas only 7 radiology reports mentioned hyperdense MCA sign (36% versus 14%; *P* < 0.0198). Hyperdense basilar sign was seen in 1 patient using Stroke Windows, whereas the final radiology report detected none (*P* < 1.0). Similarly, sulcal effacement was seen in 7 patients by the resident using Stroke Windows, whereas none were reported in the final radiology report (*P* < 0.0125). Basal ganglia hypodensity was reported in 7 patients by the resident and in 2 patients' final reports (14% versus 4%; *P* < 0.15). Finally, loss of grey-white differentiation was graded in 10 patients by the resident and in 2 patients by the staff radiologist (20% versus 4%; *P* < 0.027).  Figure 1 illustrates representative cases demonstrating how standard Stroke Windows may increase the diagnostic yield of NCCT in the setting of acute ischemic stroke. Chart review demonstrated a 100% correlation between both the resident and radiologists' interpretation with the final discharge diagnosis.

## 4. Discussion

NCCT is recommended by the American Heart Association as the initial modality of choice for stroke investigation [8] because of its accessibility, speed, and patient tolerance, thereby permitting the rapid triage of patients suspected of having experienced a stroke. Several studies have shown an association between baseline infarct size and final infarct volume, clinical outcome, and hemorrhagic transformation risk when using intravenous thrombolysis with tissue plasminogen activator and/or intra-arterial stroke interventions [2, 9, 10]. Additionally, studies suggest that hypoattenuation on CT is highly specific for irreversible ischemic brain damage if detection occurs within the first 6 hours. Patients without hypoattenuating brain tissue have a more favorable clinical course [11]. The absence of an identifiable infarct on initial imaging does not preclude stroke treatment in the appropriate clinical context; however, visualization of infarct confirms the clinical diagnosis and allows for a more nuanced treatment decision regarding thrombolysis and endovascular intervention to be made on the basis of the size and severity of infarct on presentation [11, 12].

In this study, we have demonstrated that the detection of EIC by NCCT is significantly improved by utilization of standard Stroke Windows and can be easily utilized by the initial providers assessing acute stroke, including the trainees. This has important implications concerning the delivery of acute stroke care, especially considering that residents are often the first clinicians to see these patients and that the majority of acute stroke thrombolysis in the United States is delivered at academic institutions [13].

This study has several important limitations. First, this is a single center study with a single neurology resident performing image interpretation. Secondly, the patients do not represent a random population, rather a group of individuals with known history of stroke or acute stroke; therefore, the blinding of the resident reviewer was not complete. This introduces bias which may have resulted in the resident reporting more positive findings than the staff radiologist. Additionally, we did not limit our study to specific vascular distribution and included all patients with NIHSS > 4, including stroke mimics (1 patient had no stroke at the time of discharge). Also, the NCCT interpretation by the senior resident using standard windows was not compared with the staff radiologists' interpretation at the same window settings to establish user-dependent variability in image interpretation. We know from previously well-established studies that the total time taken for image interpretation is an important factor in detection of EIC [14]. Our study did not take CT scan reading time into consideration. Finally, not all interpreting radiologists were subspecialty trained in Neuroradiology, which may have contributed to low rates of EIC detection but may also represent a real-world setting in regard to the radiology workforce that interprets the bulk of acute ischemic stroke CTs [15].

## 5. Conclusion

Despite some limitations, the rate of detection of EIC significantly improved with the use of Stroke Windows in the authors' experience. Further randomized studies using Stroke Windows may help in establishing a gold standard for CT interpretation in the setting of acute ischemic stroke. At this time, based on our single center experience, the authors would recommend the use of Stroke Windows by neurology trainees, stroke neurologists, radiologists, and ED physicians to improve the detection of EIC in the setting of acute ischemic stroke.

## Figures and Tables

**Table 1:** Comparison of the number of detected EICs (*N* = 50).

EIC	Number of resident-detected EICs^a^	Number of staff radiologist-detected EICs^b^	Significance
Hyperdense MCA sign	18 (36%)	7 (14%)	*P* < 0.0198
Hyperdense basilar sign	1 (2%)	0 (0%)	*P* < 1.0
Sulcal effacement	7 (14%)	0 (0%)	*P* < 0.0125
Basil ganglia hypodensity	7 (14%)	2 (4%)	*P* < 0.15
Loss of grey-white differentiation	10 (20%)	2 (4%)	*P* < 0.027

^a^EIC detected with author-established Stroke Window settings.

^b^EIC detected with standard windows settings.
